# The Role of Intersectionality in Access to Technology Among Older Adults

**DOI:** 10.1093/geroni/igab046.996

**Published:** 2021-12-17

**Authors:** Zainab Suntai, Susanny Beltran

**Affiliations:** 1 University of Alabama, Tuscaloosa, Alabama, United States; 2 University of Central Florida, Orlando, Florida, United States

## Abstract

In the era of COVID-19, technology has become a primary means of connecting with the world while maintaining physical distance, which is crucial for older adults who are at disproportionately high risk of infection and death. Throughout the pandemic, there has been increased emphasis on using telehealth to access medical and mental health care, and technology (e.g., apps, social media, video calls) for social interactions/communication to mitigate loneliness/isolation. Thus, COVID-19 has increased the need for older adults to access technology, and widened disparities experienced by those with limited access. This study used data from the 2018 National Health and Aging Trends Study, an annual longitudinal panel survey of Medicare beneficiaries aged 65+ in the U.S, to explore the association between the interaction of race/ethnicity and sex, and access to both a working cell phone and laptop/computer. Chi-square tests and multivariable logistic regressions were conducted. The sample (N=2,442) was 83.7% white, 8.5% Black, and 7.8% Hispanic. After accounting for other explanatory variables, logistic regression analysis indicated significantly higher odds of not having both a working cell phone or computer/laptop among White women (OR=1.518, CI=.1.510-1.527), Black men (OR=.1.741, CI= 1.720-1.763), Black women (OR=2.567, CI= 2.545-2.589), Hispanic men (OR=1.036, CI=1.022-1.050), and Hispanic women (OR=2.265, CI=.2.243-2.287) compared to White men. Overall, Hispanic and Black women were the least likely to have access to technology compared to other groups. Addressing technological equity remains a need. Future research should consider how the provision of devices along with technological literary programs can improve well-being among BIPOC women.

